# Urinary tract infections and risk of preterm birth: a systematic review and meta-analysis

**DOI:** 10.1590/S1678-9946202466054

**Published:** 2024-09-06

**Authors:** Erping Wang, Peng Tang, Chen Chen

**Affiliations:** 1 The First People’s Hospital of Linping District, Urology Surgery, Hangzhou, Zhejiang Province, China

**Keywords:** Urinary tract infections, Preterm birth, Pregnancy, Meta-analysis

## Abstract

This systematic review and meta-analysis assessed the association between urinary tract infections (UTIs) during pregnancy and the risk of preterm birth (PTB). We searched multiple databases for relevant observational studies, categorizing them as UTI-based (comparing PTB incidence in women with and without UTIs) or PTB-based (comparing UTI prevalence in women with and without PTB). Using a random-effects model in Stata software version 17.0, we estimated pooled and adjusted odds ratios (ORs) with 95% confidence intervals (CIs), and performed subgroup, sensitivity, and cumulative analyses to explore heterogeneity. In total, 30 studies comprising 32 datasets were included, involving a total of 249,810 cases and 2,626,985 healthy controls. The meta-analysis revealed a significant positive association between UTIs during pregnancy and PTB occurrence (OR, 1.92; 95% CI, 1.62–2.27). A sub-group analysis based on studies, the participants showed significant association in both PTB-based (OR, 2.01; 95% CI, 1.58–2.56) and UTI-based studies (OR, 1.79; 95% CI, 1.42–2.26). However, Egger’s test indicated the presence of publication bias (p=0.020), and substantial heterogeneity was observed across the included studies (*I* ^2^=96.6; p< 0.001). These findings emphasize the critical importance of early detection and effective management of UTIs in pregnant women to reduce the risk of PTB and its associated adverse outcomes. While the results highlight a robust link between UTIs during pregnancy and PTB risk, the potential influence of publication bias and substantial heterogeneity should be considered to interpret these findings. Further research is needed to better understand the underlying mechanisms and to develop targeted interventions for high-risk pregnant women.

## INTRODUCTION

Preterm birth (PTB), characterized by the birth of a baby before completing 37 weeks of gestation, persists as a substantial public health issue on a global scale. In 2020, the estimated prevalence of preterm births worldwide stood at 9.9% (with a confidence interval of 9.1–11.2%), accounting for approximately 13.4 million live births occurring prematurely (ranging from 12.3 to 15.2 million)^1^. Despite advancements in prenatal and newborn healthcare, PTB persists as a significant cause of both mortality and morbidity among newborns, contributing to roughly 0.9 million deaths annually across the globe^2^. The burden of PTB extends beyond neonatal outcomes, often resulting in long-term health complications, including neuro-developmental disabilities, respiratory distress syndrome, cerebral palsy, and other chronic diseases^3,4^. The cause of PTB is multifaceted, encompassing an intricate interaction of genetic, environmental, and sociodemographic factors^5^. Among the various risk factors implicated in PTB, infections, mainly those of the amniotic fluid and lower genital tract, as well as inflammations during pregnancy have garnered significant attention due to their potential role in triggering preterm labor and delivery^6,7^. Available evidence suggests that microbial invasion of the amniotic cavity, maternal genitourinary infections, and systemic inflammatory responses may disrupt the delicate balance of maternal-fetal immune tolerance, culminating in preterm birth^8^.

Urinary tract infections (UTIs) are among the prevalent bacterial infections encountered during pregnancy, impacting an estimated 2–15% of pregnant women worldwide^9,10^. UTIs encompass a spectrum of microbial colonization and invasion within the urinary tract, ranging from asymptomatic bacteriuria to acute pyelonephritis^11^. The association between UTIs and adverse pregnancy outcomes, such as preeclampsia, PTB, intrauterine growth restriction, and low birth weight, has long been recognized in clinical practice^9^. However, the precise mechanisms underlying this association remain incompletely understood^9^. Emerging evidence suggests that UTIs may contribute to the pathogenesis of PTB via several potential mechanisms. First, ascending bacterial colonization from the lower urinary tract to the upper reproductive organs, including the uterus and fetal membranes, can lead to localized inflammation and subsequent preterm labor^12^. Additionally, UTIs may trigger systemic inflammatory responses characterized by the release of proinflammatory cytokines and chemokines, further promoting uterine contractility and cervical ripening^13,14^.

Despite the biological plausibility of UTIs as a risk factor for PTB, the existing literature has yielded conflicting findings, requiring a comprehensive synthesis of available evidence. Hence, this systematic review and meta-analysis aims to evaluate the association between UTIs and the risk of PTB. By synthesizing data from published studies, we seek to elucidate the magnitude of this association, explore potential sources of heterogeneity, and identify gaps in knowledge. Ultimately, our findings may inform clinical practice guidelines and public health strategies aimed at mitigating the burden of PTB associated with UTIs.

## MATERIALS AND METHODS

### Search strategy and study eligibility criteria

This systematic review and meta-analysis study was performed and reported according to the MOOSE^15^ and PRISMA^16^ guidelines, respectively. The PubMed, Scopus, Embase, and Web of Science databases were searched for eligible studies published from inception to April 10^th^, 2024, using the following keywords: (“Urinary Tract Infection” OR “UTI” OR “cystitis” OR “pyelonephritis” OR “bacteriuria”) AND (“Preterm Birth” OR “Premature Birth” OR “preterm labor” OR “preterm neonates” OR “Maternal outcome” OR “pregnancy complication” OR “adverse pregnancy outcome”) (Supplementary Figure S1). A manual search of reference lists was also conducted in pertinent review articles and identified papers. The Google Scholar was also explored up to the first 40 pages to identify any supplementary studies or grey literature. This study imposed no language or geographical limitations during the search process. The Google Translate online tool was used to verify non-English articles. The EndNote X software (version 8.0, Thomson Reuters, California, USA) was used for record management, deduplication, and initial screening of abstracts and titles. We included peer-reviewed observational studies, such as cross-sectional surveys, cohort studies, or case-control studies, which enrolled at least 30 pregnant women (aged 15 years or older). These studies compared the incidence of PTB in pregnant women who tested positive for UTIs and those who tested negative (referred to as UTI-based studies), or compared the prevalence of UTIs in pregnant women with and without PTB (referred to as PTB-based studies). We excluded studies that duplicated datasets from other studies, included patients with known UTIs at the beginning of the study without subsequent follow-up for PTB, studies focusing solely on PTB infants without extractable data on maternal characteristics, and/or studies lacking a control group. Additionally, case reports, case series, literature reviews, systematic reviews, and other types of studies lacking original data were also excluded.

### Data extraction and quality assessment

Data from eligible studies were independently extracted and rechecked by all investigators using a pre-designed data extraction form in Microsoft Excel 2013 (Microsoft Corporation, Redmond, Washington, USA). The data were then corroborated, and any discrepancies were resolved by discussion. The following data were extracted from the studies: author details, year of publication, study period, geographic area of the study (country, World Health Organization region), study design, study groups, sample size, outcome of interest (incidence of PTB or prevalence of UTI), adjusted relative risks (RRs) or odds ratio (ORs) and 95% confidence intervals (CIs). Study quality was assessed using the Newcastle–Ottawa scale (NOS) designed for non-randomized studies^17^. The NOS adapted for cross-sectional studies assesses quality based on sample representativeness, sample size, handling of non-respondents, and exposure ascertainment (maximum of five scores), group comparability (maximum of two scores), and outcome assessment (maximum of three scores). For case-control studies, quality assessment focuses on study group selection, case definition, representativeness of cases, control selection, and definition (maximum of four scores), group comparability (maximum of two scores), and exposure ascertainment (maximum of three scores). In cohort studies, quality evaluation considers the selection of study groups, cohort representativeness, exposure ascertainment, and absence of the outcome at the study’s onset (maximum of four scores), cohort comparability based on design or analysis (maximum of two scores), and outcome assessment (maximum of three scores). Studies with higher number of stars held higher quality and lower risk of bias.

### Data synthesis

Statistical analyses were conducted using Stata software (version 17.0; Stata Corporation, College Station, TX, USA). Given that most of the included studies presented effect sizes as odds ratios (ORs), the association between UTIs and PTB was assessed by estimating pooled ORs and corresponding 95% confidence intervals (CIs). Pooled results were generated using the restricted maximum-likelihood random-effects model (REM), which accounts for both within-study and between-study variations. Forest plots were employed to visualize the dispersion of observed ORs. For instances in which studies showed zero cells, a continuity correction of 0.5 was implemented. Weighted pooled adjusted ORs were derived from studies reporting adjusted ORs exclusively. Subgroup analyses were conducted to explore potential sources of heterogeneity considering type of study, such as income level. Heterogeneity among studies was assessed using *I* ^2^statistics. Sensitivity analysis was performed to assess the influence of individual studies on the results. To investigate potential publication bias and assess symmetry among included studies, a contour-enhanced funnel plot and Egger’s test were applied. Statistical significance was set at p < 0.05.

## RESULTS

**Figure 1:**
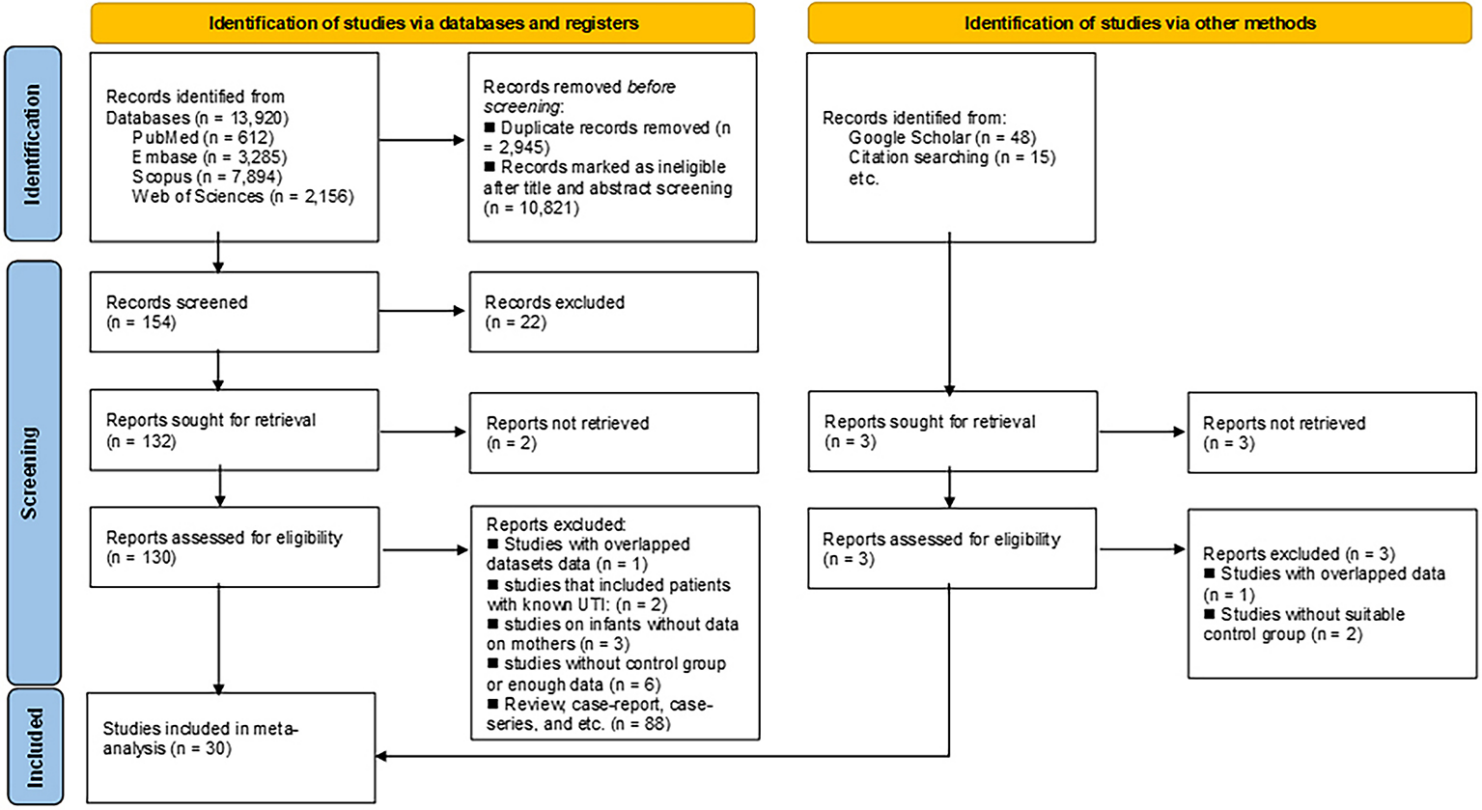
PRISMA flow diagram for study-selection for the meta-analysis.

Our initial search across databases yielded 13,920 potentially relevant publications. Following duplicate removal, 2,945 duplicates were excluded, leaving 10,821 publications for title and abstract screening. Additionally, we identified 63 studies via Google Scholar and manual searches. After thorough screening, 133 articles underwent in-depth review for eligibility, resulting in 30 studies^18-47^ comprising 32 datasets for inclusion in the meta-analysis (Figure 1). The selected studies spanned the years 2007 to 2024 and encompassed a total of 249,810 cases and 2,626,985 healthy controls. These studies were conducted in 17 countries, including Bangladesh, Brazil, Egypt, Ethiopia, Hungary, India, Iran, Israel, Kenya, Mexico, Netherlands, Norway, Romania, Rwanda, Taiwan, United Arab Emirates, and USA. Among the included datasets, 21 were PTB-based and 11 were UTI-based studies. Study designs varied, with 15 case-control, nine retrospective cohort, seven prospective cohort, and one cross-sectional study. Quality assessment using the NOS categorized 26 datasets as having low risk (NOS total score ≥ 7) and six as having moderate risk (NOS total score 4–6) of bias. Tables 1 and 2 present the main characteristics of eligible PTB-based and UTI-based studies, respectively.

**Table 1:** Main characteristic of studies that assessed prevalence of UTIs in pregnant women with and without PTB.

Article	Study duration	Country	Study design	Pregnant women with PTB	UTI-infected	Pregnant women without PTB	UTI-infected	Quality Score
Agger *et al.* ^18^	2008–2011	USA	PC	54	15	622	68	9
Alijahan *et al*.^19^	2010–2011	Iran	CC	332	61	569	71	9
Alijahan *et al*.^19^	2010–2011	Iran	CC	266	37	506	45	9
Assefa *et al*.^20^	2021	Ethiopia	CC	79	15	237	19	9
Escobar-Padilla *et al*.^21^	2011–2012	Mexico	CC	344	119	344	88	8
Bernardo *et al*.^22^	2010–2011	Brazil	PC	74	10	1214	91	9
Fetene *et al*.^23^	2020	Ethiopia	CC	135	44	270	30	9
Baer *et al*.^24^	2011–2017	USA	RC	186,610	13351	2,061,528	87,341	9
Kamel *et al*.^25^	2016–2017	Egypt	CC	120	5	40	1	5
Ibrahim *et al*.^26^	2020–2021	Egypt	CC	50	4	50	7	5
Kunpalin *et al*.^27^	2005–2015	UK	RC	86	12	165	7	7
Hosny *et al*.^28^	2009–2010	Egypt	CC	72	58	45	42	6
Morken *et al*.^29^	1999–2008	Norway	CC	1,758	179	64,173	6,289	6
Nsereko *et al*.^30^	2017	Rwanda	PC	37	16	330	29	8
Pérez Molina *et al*.^31^	2005–2006	Mexico	CC	92	17	92	7	7
Pérez-Molina *et al*.^32^	2011–2012	Mexico	CC	343	82	686	113	7
Sureshbabu *et al*.^33^	2019	India	CC	191	23	200	7	9
Wagura *et al*.^34^	2013	Kenya	CS	59	28	263	84	8
Wakeyo *et al*.^35^	2018	Ethiopia	CC	61	11	183	13	8
Vidhyalakshmi *et al*.^36^	2013–2015	India	CC	50	11	50	5	7
Tellapragada *et al*.^37^	2011–2014	India	PC	54	3	656	20	8

UK = United Kingdom; PC = Prospective cohort; RC = Retrospective Cohort; CC = cross-sectional; CS = cross-sectional, UTIs = urinary tract infections; PTB = preterm birth.

**Table 2:** Main characteristic of studies that assessed incidence of PTB in pregnant women with and without UTIs.

Article	Study duration	Country	Study design	UTIs+ pregnant women	Incidence of PTB	UTIs- pregnant women	Incidence of PTB	Quality Score
Anderson *et al.* ^38^	2002–2004	USA	RC	203	42	220	35	8
Chen *et al*.^39^	2000–2003	Taiwan	RC	41,105	3163	42,742	3,062	9
Ba´nhidy *et al*.^40^		Hungary	CC	2,188	228	35,963	3,268	9
Balachandran *et al*.^41^	2018	UAE	RC	518	99	329	40	7
Baer *et al*.^24^	2011–2017	USA	RC	4,492	356	21,858	1,231	9
Mazor-Dray *et al*.^42^	1988–2007	Israel	RC	4,742	716	194,351	15,160	7
Radu *et al*.^43^	2014–2023	Romania	RC	183	33	183	10	8
Sheiner *et al*.^44^	1988–2007	Israel	RC	4,890	650	194,203	147,60	8
Schuster *et al*.^45^	2014–2016	Netherlands	PC	25	7	324	19	8
Anayet Ullah *et al*.^46^		Bangladesh	PC	134	44	134	24	6
Werter *et al*.^47^	2011–2013	Netherlands	PC	463	56	4,455	229	9

UAE = United Arab Emirates; PC = Prospective cohort; RC = Retrospective Cohort; CC = cross-sectional; UTIs = urinary tract infections; PTB = preterm birth.

### Results of overall meta-analysis

**Figure 2:**
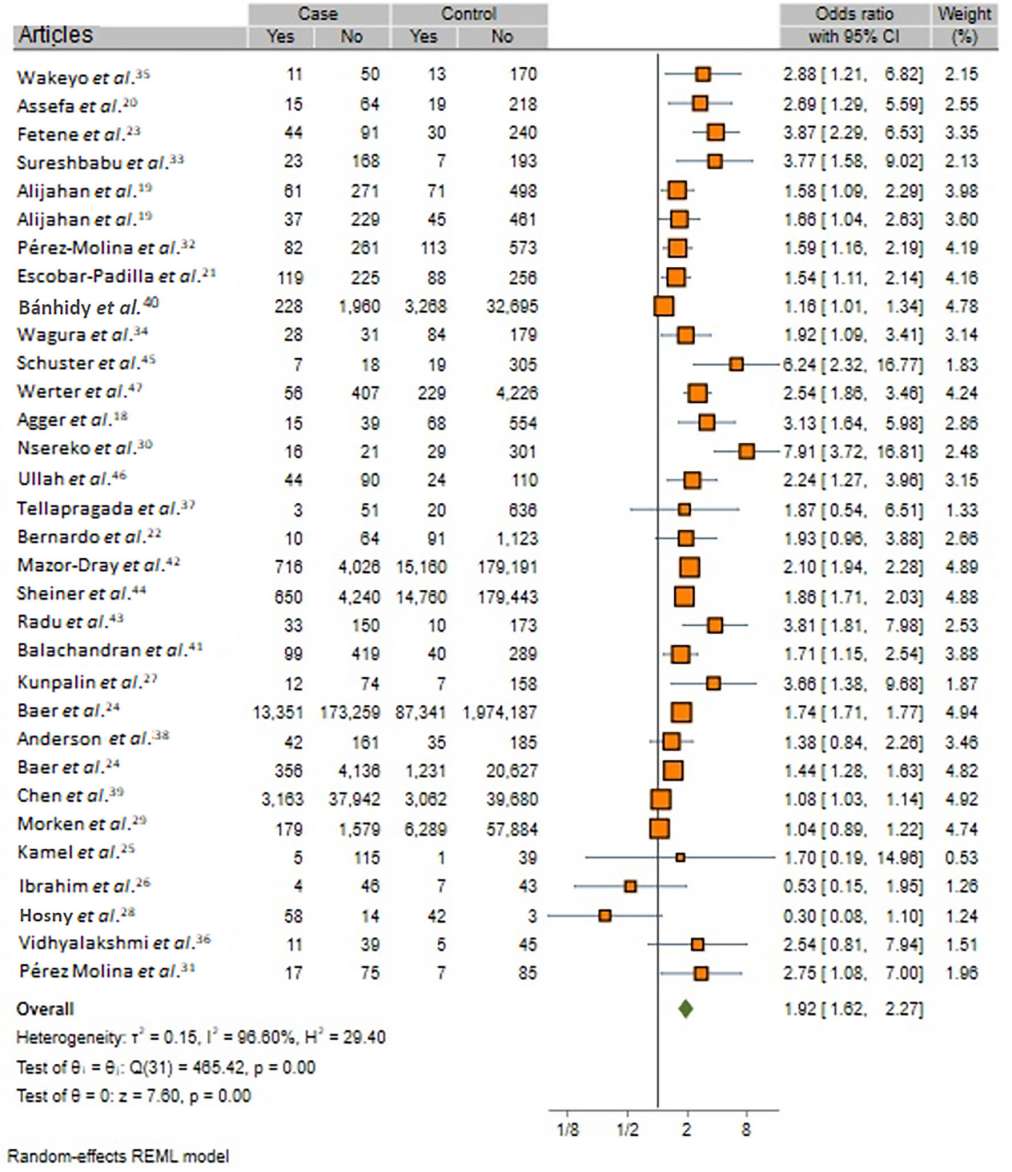
Forest plots for overall meta-analysis of studies reporting effect of exposure to UTIs during pregnancy on risk of PTB.

**Figure 3:**
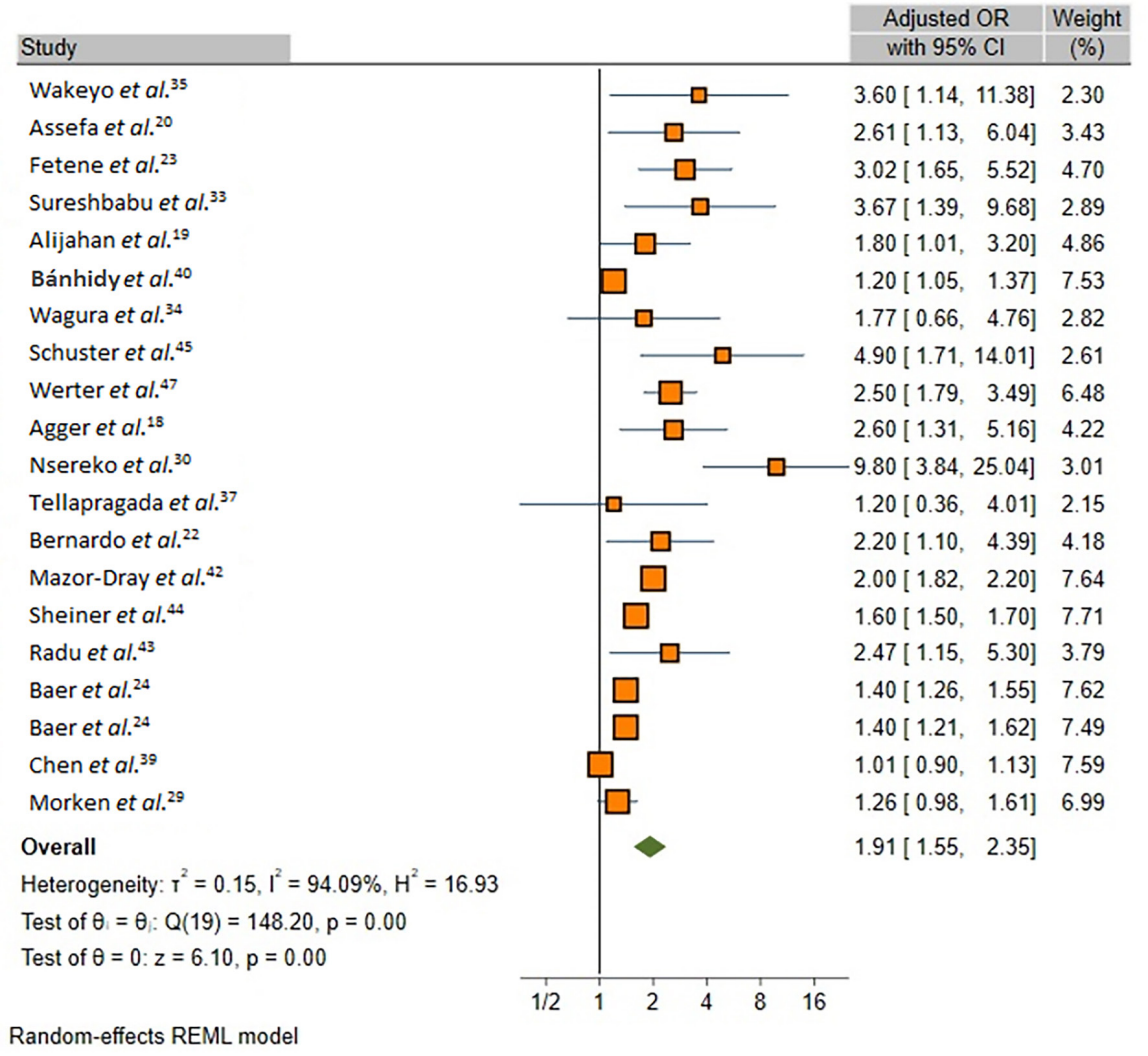
Forest plots for overall meta-analysis of studies that reported adjusted odds ratios on effect of UTIs during pregnancy on risk of PTB.

As depicted in Figure 2, the overall meta-analysis revealed a statistically significant positive association between UTIs during pregnancy and occurrence of PTB (OR, 1.92; 95% CI, 1.62–2.27; *I* ^2^=96.6; p< 0.001). Further analysis using REM on adjusted ORs also confirmed a significant positive association between UTIs and PTB development (OR, 1.91; 95% CI, 1.55–2.35; *I* ^2^ = 94.09%; Figure 3). Assessment of “small-study effects” via funnel plots, as depicted in Supplementary Figure S2, suggested symmetry without indications of publication bias; however, Egger’s test indicated evidence of publication bias (Egger’s regression intercept: 0.96, p= 0.020).

### Sub-group meta-analysis based on PTB-based datasets

A subgroup meta-analysis involving 21 eligible datasets encompassing 190,867 pregnant women with PTB and 2,132,223 without PTB showcased UTIs prevalence rates of 20.61% (95% CI, 15.40–26.34%; 14,101/190,867) and 11.53% (95% CI, 9.10–14.19%; 94,377/2,132,223), respectively (Table 3). REM revealed a significantly higher prevalence of UTIs in pregnant women with PTB (OR, 2.01; 95% CI, 1.58–2.56; *I* ^2^=87.04%), indicating a substantial positive association between UTIs during pregnancy and PTB occurrence. This association was further confirmed by adjusted ORs (OR, 2.21; 95% CI, 1.61–3.03; *I* ^2^ = 76.74%).

**Table 3:** Sub-group analyses of the pooled odds ratios for the association between UTIs during pregnancy and PTB.

Variables	Datasets (n)	Pooled prevalence of outcome in cases % (95% CI)	Pooled prevalence of outcome in controls % (95% CI)	Odds ratios (95% CI)	Heterogeneity (I2%)		
					I^2^	Q	p-value
**Type of studies**							
PTB-based	21	20.61 (15.40–26.34)	11.53 (9.10–14.19)	2.01 (1.58–2.56)	87.04	89.25	<0.001
UTI-based	11	14.72 (11.82–17.87)	7.44 (6.82–8.10)	1.79 (1.42–2.26)	96.31	277.24	<0.001
**Study design**							
Case-control	15	21.10 (16.04–26.16)	13.57 (11.64–15.50)	1.68 (1.28–2.20)	81.80	54.62	<0.001
Retrospective cohort	8	13.85 (10.79–16.90)	7.28 (5.47–9.09)	1.80 (1.58–2.06)	88.35	38.71	<0.001
Prospective cohort	7	21.63 (13.09–30.16)	7.44 (5.36–9.51)	3.05 (2.10–4.44)	55.42	12.39	0.054
Cross-sectional	2	7.71 (7.45–7.97)	7.21 (6.97–7.45)	1.34 (0.77–2.31)	74.17	3.87	0.049
**Income levels**							
High	14	11.99 (10.66–13.31)	7.63 (6.21–9.08)	1.78 (1.43–2.22)	98.31	419	<0.001
Lower middle	10	23.94 (13.47–34.40)	19.48 (10.23–28.73)	1.72 (1.39–2.13)	0	14.87	0.095
Upper middle	4	23.02 (14.28–31.75)	14.23 (6.51–21.96)	1.64 (1.33–2.03)	0	1.56	0.668
Low	4	27.05 (16.96–37.14)	8.73 (7.01–10.46)	3.95 (2.55–6.11)	4.84	35.92	0.184
**Study quality**							
High	26	15.63 (14.25–17.01)	8.99 (7.93–10.05)	2.02 (1.71–2.39)	96.57	420.15	<0.001
Moderate	6	23.45 (9.75–37.15)	22.79 (3.81–41.76)	1.14 (0.58–2.22)	64.79	11.21	0.047

Sub-group meta-analysis based on UTI-based datasets

Considering 11 eligible studies involving 58,943 pregnant women with UTIs and 494,762 without UTIs, the pooled occurrence of PTB was 14.72% (95% CI, 11.82-17.87%; 5,394/58,943) and 7.44% (95% CI, 6.82-8.10%; 37,838/494,762), respectively (Table 3). REM indicated a significantly higher incidence of PTB in pregnant women with UTIs (OR, 1.79; 95% CI, 1.42-2.26; *I* ^2^=96.31%), highlighting a robust positive association between UTIs during pregnancy and PTB occurrence. Adjusted ORs further supported this association (OR, 1.65; 95% CI, 1.27–2.15; *I* ^2^ = 96.13%).

### Sub-group meta-analyses based on characteristics of studies

Subgroup analyses based on study design demonstrated a significant association between UTIs and PTB across prospective cohorts (OR, 3.05; 95% CI, 2.1–4.44), retrospective cohorts (OR, 1.80; 95% CI, 1.58–2.06), and case-control studies (OR, 1.62; 95% CI, 1.28–2.20). However, non-significant associations were observed in cross-sectional studies (OR, 1.34; 95% CI, 0.77–2.31). Notably, this association remained significant across all subgroups based on the income levels of countries (Table 3). Additionally, subgroup analysis based on study quality indicated a significant association between UTIs and PTB in high-quality studies (OR, 2.02; 95% CI, 1.71–2.39), whereas no significant association was observed in studies with moderate quality (OR, 1.14; 95% CI, 0.58–2.22; Table 3).

### Sensitivity and cumulative analysis

A sensitivity analysis was conducted to assess the impact of individual studies on the overall pooled effect sizes. Results indicated that omitting any single study did not substantially alter the overall pooled OR (Supplementary Figure S3), indicating the robustness of the findings. Cumulative meta-analysis suggested a progressively increasing association, which remained statistically significant as evidence accumulated (Supplementary Figure S4).

## DISCUSSION

Questions persist regarding the association between maternal UTIs and preterm birth. There is no conclusive evidence, and the exact nature of this association—whether causal, confounded, or spurious—remains unclear. Our research holds significance, as UTIs commonly affect pregnant women and are easily diagnosable and treatable. Identifying a link between UTIs during pregnancy and PTB could inform interventions for early detection and treatment, addressing a significant complication in pregnant women. Our findings, derived from this comprehensive meta-analysis encompassing approximately 2.9 million participants, underscore a robust association between maternal UTIs during pregnancy and the occurrence of PTB. This significant association persisted across most subgroup analyses, as well as in sensitivity and cumulative analyses, indicating the robustness of our results. Moreover, subgroup analysis focusing on prospective cohorts revealed an even more robust positive association (OR, 3.05) with lower heterogeneity, underscoring the reliability of our findings.

Our findings corroborate and extend the existing evidence regarding the association between UTIs during pregnancy and adverse pregnancy outcomes. Several previous meta-analyses and observational studies have reported similar findings, highlighting the significant impact of UTIs on maternal and neonatal health^48-51^. For example, a meta-analysis by Yan *et al*.^48^ found a pooled OR of 1.31 for the association between UTIs and preeclampsia. Similarly, a systematic review by Wulandari *et al*.^51^ reported 1.54 times increase in the risk of low birth weight among infants of pregnant women with UTIs compared to those without UTIs. Additionally, Smaill and Vazquez^52^ investigated the association between UTIs and abortion, and reported an increased risk of abortion among pregnant women with UTIs. These findings, consistent with our results, underscore the robustness of the association between UTIs and adverse pregnancy outcomes across diverse populations and settings.

Although not fully understood, UTIs during pregnancy have been implicated in the pathogenesis of PTB by several interconnected pathophysiological mechanisms^8^. The ascending route of infection from the lower urinary tract to the upper reproductive organs, including the uterus and fetal membranes, is one of the primary pathways by which UTIs can trigger PTB. Bacterial colonization of these anatomical sites can lead to local inflammation and activation of the maternal-fetal immune response, culminating in the production of proinflammatory cytokines, chemokines, and prostaglandins^53^. These inflammatory mediators can stimulate uterine contractions, cervical ripening, and ultimately premature rupture of membranes, precipitating preterm birth^8,54^. Moreover, UTIs can induce systemic maternal inflammation, characterized by the release of inflammatory cytokines into the bloodstream. This systemic inflammatory response may contribute to uterine irritability, vascular dysfunction, and placental insufficiency, further increasing the risk of preterm labor and delivery. Additionally, chronic or recurrent UTIs during pregnancy can lead to persistent inflammation and tissue damage in the maternal urinary tract, exacerbating the risk of adverse pregnancy outcomes, including PTB^53,54^. Moreover, emerging evidence suggests that specific microbial factors associated with UTIs, such as virulence factors and biofilm formation, may play a crucial role in modulating the maternal immune response and promoting preterm labor. Certain uropathogens, including *Escherichia coli* and Group B *Streptococcus*, have been implicated in the pathogenesis of PTB by triggering inflammatory cascades and disrupting the maternal-fetal immune tolerance^55,56^. In summary, the association between UTIs during pregnancy and the development of preterm birth is multifactorial and involves complex interactions between microbial colonization, local and systemic inflammation, immune activation, and uterine contractility. Understanding these pathophysiological mechanisms is essential for developing targeted strategies for the prevention and management of UTIs in pregnant women to reduce the burden of preterm birth and its associated complications.

While our meta-analysis provides valuable insights into the association between UTIs during pregnancy and the risk of PTB, several limitations should be considered when interpreting the findings. Firstly, the included studies varied in terms of study design, sample size, geographic location, and methodological quality. Heterogeneity among studies is inevitable in meta-analyses^57,58^, and despite our efforts to address it via subgroup analyses and sensitivity analyses, residual heterogeneity may still exist. The diversity in study characteristics may affect the generalizability of our results to different populations or settings. Furthermore, several variables may contribute to the observed heterogeneity, including the clinical subtype of PTB (spontaneous or medically induced)^59^ and subtypes of spontaneous PTB (with or without premature rupture of membrane [PROM]), as PTB with PROM has a greater association with infections^5^. Additionally, the type of infections (e.g., asymptomatic bacteriuria or pyelonephritis) and the diagnostic methods used may also be sources of heterogeneity. Despite our efforts to extract relevant data, most studies included did not provide extractable data on these variables for use in the meta-analysis. Understanding these factors is crucial for interpreting the heterogeneity and enhancing the applicability of our findings to various clinical scenarios. Secondly, most studies included relied on retrospective data collection or medical record reviews, which are subject to inherent biases and limitations. Retrospective studies may suffer from recall bias, misclassification of exposure or outcome, and incomplete data documentation. Thirdly, the presence of publication bias, as suggested by Egger’s test, highlights a critical aspect of our meta-analysis. This publication bias indicates that our meta-analysis might be influenced by selective reporting of studies, in which smaller studies showing negative or non-significant results are less likely to be published. This bias is a critical aspect to consider, as it may overestimate the true association between UTIs and PTB. Although our study funnel plot demonstrated symmetry, suggesting minimal bias, the Egger’s test underscores the need for caution in interpreting the pooled estimates. The methodological rigor employed in this study, such as the use of a comprehensive search strategy, inclusion of a large number of studies, and subgroup analyses, mitigates some effects of publication bias. However, the potential for bias remains. The sources of bias in our meta-analysis could stem from varying study designs, geographical locations, and publication standards across different journals. To further contextualize our findings, we compared the degree of publication bias reported in other meta-analyses related to pregnancy outcomes. For instance, a meta-analysis by Wulandari *et al.* ^51^ on UTIs and low birth weight reported similar challenges with publication bias. Acknowledging and addressing the limitations posed by potential publication bias is essential for accurate interpretation in future studies. Further meta-analyses on this subject should aim to include unpublished studies and data from non-English publications to provide a more comprehensive assessment and further elucidate the underlying mechanisms linking UTIs to PTB. Fourthly, factors such as maternal age, parity, socioeconomic status, smoking status, and comorbidities could confound the association between UTIs and PTB but were not consistently adjusted in all included studies. Additionally, subgroup analyses based on these confounders were limited by availability of data across studies. Finally, while meta-analyses provide pooled effect estimates, they cannot establish causality. The observed association between UTIs and preterm birth may be influenced by unmeasured or unknown confounders, as well as reverse causation. Longitudinal prospective studies and randomized controlled trials are warranted to further elucidate the causal relationship and underlying mechanisms between UTIs during pregnancy and preterm birth.

## CONCLUSION

In conclusion, our meta-analysis highlights a significant association between UTIs during pregnancy and the risk of PTB. Despite advancements in obstetric care, PTB remains a global health concern. Early detection and treatment of UTIs in pregnant women are essential for mitigating the risk of PTB. Future research should focus on prospective studies and randomized controlled trials to better understand the underlying mechanisms and develop targeted interventions. Our findings underscore the importance of routine screening and prompt management of UTIs during pregnancy to improve maternal and neonatal outcomes worldwide. Due to the substantial heterogeneity between studies, as well as publication bias identified, we strongly suggest well-designed and -controlled studies in the future to rigorously assess the association between UTI during pregnancy and PTB.

## Data Availability

https://doi.org/10.48331/scielodata.N6A5AT
